# Editorial: Emerging talents in human neuroscience: psychophysiology and cognitive neuroscience 2025

**DOI:** 10.3389/fnhum.2026.2016840

**Published:** 2026-10-29

**Authors:** Valentina Bruno, Mariano D'Angelo, Mattia Galigani, Manuel Mello, Gaia Risso

**Affiliations:** 1 Manibus Lab, Psychology Department, University of Turin, Turin, Italy; 2 Department of Neuroscience, Karolinska Institutet, Stockholm, Sweden; 3 Institut des Sciences Cognitives—Marc Jeannerod, UMR5229, Centre National de La Recherche Scientifique (CNRS) and Université Claude Bernard Lyon 1, Bron, France; 4 Institute of Health, School of Health Sciences, HES-SO Valais-Wallis, Sion, Switzerland; 5 The Sense Innovation and Research Center, Sion/Lausanne, Switzerland

**Keywords:** body representation, cognitive neuroscience, emotional wellbeing, lifespan development, multisensory integration, neural biomarkers, neuromodulation, psychophysiology

This Research Topic highlights studies led by emerging researchers, including undergraduate and graduate students and researchers within the first years of their academic careers, showcasing the originality and scientific rigor of the next generation of scientists in psychophysiology and cognitive neuroscience. The works presented here cover the human lifespan and, although addressing diverse research questions, they can be organized around three complementary perspectives: the adaptive construction of bodily experience, the identification of neural markers of developmental and cognitive vulnerability, and the development of innovative approaches for modulating brain function, cognition, and emotional wellbeing.

The first perspective concerns the adaptive construction of bodily experience across the lifespan and under different physiological and clinical conditions. Bodily self-representation is increasingly conceptualized as a dynamic process grounded in the integration of multisensory bodily signals and their interaction with internal models of the body (Tsakiris, 2010; Serino et al., 2013; Blanke et al., 2015). Building on psychophysical and computational approaches previously employed to investigate body representation and multisensory processing (Risso et al., 2025; Mastria et al., 2025), Martinelli et al. examined how the sense of body ownership changes with aging (Risso et al., 2025) by using a continuous life-span approach, from adolescence to old age. They showed that older age is associated with greater reliance on visual information, possibly compensating for the gradual decline in proprioceptive precision and helping to maintain a coherent representation of the body. In parallel, Gentili et al. provide a systematic mapping of the last 15 years of research on anorexia nervosa, reporting a crucial shift from studies focused primarily on perceived body image and aesthetic evaluation to more complex paradigms examining multisensory integration and the implicit body schema. This shift reflects a broader reconceptualization of anorexia nervosa as involving not only disturbances in the conscious evaluation of body appearance, but also alterations in the multisensory processes underlying bodily self-representation and supporting the development of interventions that directly target body representations through innovative technology-assisted approaches (Meregalli et al., 2026; Risso and Bassolino, 2022). Complementing these perspectives, Bartolini et al. provide a mini-review of interceptive skills in visually impaired children, integrating evidence from both pediatric and adult populations. By examining how visual information contributes to the multisensory processes supporting goal-directed interactions between the body and the environment, their work reports how the absence of early visual input may constrain the development of motor coordination and social interaction. They further emphasize the need for more ecologically valid, multisensory, and longitudinal research, while discussing the potential of emerging technologies, such as virtual reality, to support accessible, engaging, and individualized rehabilitative interventions. Together, these studies show the dynamic and multisensory nature of bodily experience, demonstrating how body representation continuously adapts across development, aging, and clinical conditions.

The second perspective focuses on the identification of neural markers of developmental and cognitive vulnerability. Petri et al. provide a narrative review showing the complementary role of oscillatory and aperiodic EEG activity in characterizing early brain development, building on methodological advances that allow these spectral components to be parameterized separately (Donoghue et al., 2020). By discussing methodological advances, developmental changes, and alterations associated with neurodevelopmental disorders, they emphasize the potential of spectral EEG analyses to advance both basic and clinical neuroscience. Extending this biomarker perspective to adulthood, Bi et al. explored the relationship between hippocampal subfield atrophy and working memory deficits in middle-aged and older cancer patients. Their results revealed reduced volumes in specific hippocampal subfields, particularly CA3 and CA4, while volumes of the hippocampal head and molecular layer were positively associated with working memory performance in cancer patients, in line with evidence that distinct hippocampal subregions are differentially vulnerable across disease and aging (Small et al., 2011). Finally, Li et al. demonstrated that auricular acupressure and massage can enhance cognitive functions in night shift nursing staff. Changes in P300 parameters, a well-established electrophysiological index associated with attentional and memory-related processes (Polich, 2007), provided objective evidence supporting the cognitive effects of the intervention. These findings point to a non-invasive and cost-effective approach to counter the cognitive impact of shift work. Across these contributions, neurophysiological and neuroanatomical markers emerge as increasingly important for understanding cognitive functioning across the lifespan and for guiding future clinical applications.

The third perspective concerns the development of original strategies for modulating brain function, cognition, and emotional wellbeing. Pic Roca et al. present a novel protocol to modulate delta oscillations during general anesthesia using closed-loop auditory stimulation, building on evidence that phase-targeted auditory stimulation can enhance slow oscillatory activity (Ngo et al., 2013). This technique aims to stabilize the state of unconsciousness without increasing pharmacological doses, opening the possibility of safer and more personalized anesthesia. Yamamoto et al. complement this approach by showing that physiological responses to stimulation do not always translate into behavioral changes. They investigated the effects of transcutaneous auricular vagus nerve stimulation (taVNS) on response inhibition, in the context of the well-established role of the locus coeruleus-noradrenergic system in regulating cognitive performance (Aston-Jones and Cohen, 2005). Although stimulation increased levels of salivary alpha-amylase (a marker of noradrenergic activity), no improvement in response inhibition, assessed with a stop-signal task, was observed after stimulation. This suggests that noradrenaline modulation via taVNS, at least under the protocol used, may not be sufficient to enhance inhibitory control in simple tasks. Finally, Moezzi et al. offer a perspective on visual art therapy, pointing to its potential to reduce stress and anxiety by modulating activity in the dorsolateral prefrontal cortex. Their review stresses the need to use objective physiological measures (such as heart rate variability and cortisol levels) to validate the efficacy of art-based therapies in treating emotional pain, consistent with previous evidence that even a single art-making session can be associated with reductions in salivary cortisol (Kaimal et al., 2016). Overall, these studies show both the promise and the complexity of modulating brain function through new interventions, and suggest that neuromodulation and complementary therapies should be evaluated through behavioral and objective physiological measures together, paving the way toward more effective and personalized clinical applications.

The contributions gathered in *Emerging talents in Human Neuroscience: Psychophysiology and Cognitive Neuroscience 2025* illustrate the range of contemporary psychophysiology and cognitive neuroscience. The three complementary perspectives represented in this collection span the adaptive construction of bodily experience, the identification of neural markers of developmental and cognitive vulnerability, and the development of novel approaches for modulating brain function, cognition, and emotional wellbeing. Across these perspectives, behavioral, neurophysiological, neuroimaging, and computational approaches provide converging tools for addressing questions across basic and translational neuroscience. Collectively, these studies offer a glimpse into the future directions of psychophysiology and cognitive neuroscience, while also highlighting both the creativity and innovation of the next generation of scientists and the increasingly interdisciplinary field, integrating experimental rigor with clinical relevance to better understand brain function across diverse populations and contexts.
